# Editorial: Human-in-the-loop system design and control adaptation for behavior-assistant robots

**DOI:** 10.3389/fnins.2024.1418331

**Published:** 2024-05-24

**Authors:** Bi Zhang, Yuquan Leng, Benyan Huo, Ningbo Yu, Xu Jin

**Affiliations:** ^1^State Key Laboratory of Robotics, Shenyang Institute of Automation, Chinese Academy of Science (CAS), Shenyang, China; ^2^Department of Mechanical and Energy Engineering, Southern University of Science and Technology, Shenzhen, China; ^3^School of Electrical and Information Engineering, Zhengzhou University, Zhengzhou, China; ^4^College of Artificial Intelligence, Nankai University, Tianjin, China; ^5^Department of Mechanical and Aerospace Engineering, University of Kentucky, Lexington, KY, United States

**Keywords:** editorial, human-in-the-loop system, behavior-assistant robots, adaptation, safety

With the progress and development of human-robot systems, the coordination among humans, robots, and environments has become increasingly sophisticated. In this Research Topic, we focus on an important field in robotics and automation disciplines, which is defined as behavior-assistant robots [i.e., rehabilitation robots, assistive robots (Zhang et al., 2024), homecare robots (Zhao et al.) and so on]. The emergence of robot-assisted daily behaviors is gradually becoming a part of social life, which improves weak motor abilities, enhances physical functionalities, and enables various other benefits. For effective operation of a behavior-assistant robot, one successful strategy is human-in-the-loop (HIL) control architecture (Zhang et al., 2017). Integration of perception, actuation, and control technologies forms a HIL system.

Multimodal information-based pattern recognition is an important pathway to obtain human motion intention. Intelligent wheelchair is a common rehabilitation device, which is indispensable for people with limited mobility. Huang et al. utilized surface electromyography (sEMG) and pressure sensors to improve wheelchair user sitting posture and alleviate lumbar muscle fatigue. The results could provide more scientific guidance and suggestions for the daily use of wheelchairs. Lower limb rehabilitation robots, including both rigid exoskeleton (Sankai and Sakurai, 2018) and exosuit (Nuckols et al., 2021), are crucial devices for stroke or spinal cord injury patients. A key factor of rehabilitation robots is to estimate continuous joint motions based on sEMG and thus to ensure unhindered human-robot interaction. Liu et al. proposed a multilayer CNN-LSTM network incorporating the self-attention mechanism, which extracted and learnt the periodic and trend characteristics of sEMG signals, and realized the accurate autoregressive prediction of human motion information. The results had shown that it is applicable for both healthy and hemiplegic individuals under non-ideal sEMG conditions. Deformable registration also plays a fundamental role for image-assisted analysis. Li A. et al. proposed a novel registration network called multi-scale feature extraction-integration network, which utilized global and local features in images. This algorithm has the potential to predict displacement fields with high accuracy. These bio-machine interfaces expand information dimensions, improve modeling precision, and promote connections with the human nervous system, which are crucial for enhancing robot intelligence technologies.

Safety of HIL systems is a considerable gap to be bridged before advanced technologies could be widely applied in clinical rehabilitation settings. Rehabilitation assessment provides a basis for formulating treatment programs and judging effects. It is conducted based on the level of function, degree of damage, and recovery of stroke patients; however, it is subjective, time-consuming, and non-uniform for clinicians. Bai et al. proposed an automatic rehabilitation assessment method for hemiplegic upper limb motor function, which could allow for automated measurement of 30 items within the Fugl-Meyer scale. Experiments with 17 participants had demonstrated a significant correlation between the results of the automated assessment system and those of the physician's assessment. For real-time human-robot interaction, a monitoring system incorporating supervised loop is anticipated to effectively counter uncertainties arising from recognition inaccuracies. Zhu et al. proposed a novel exoskeleton robotic system with remote monitoring function for lower limb rehabilitation, the force and motion of which were analyzed in detail to implement closed-loop control. The results had shown that the exoskeleton robot has satisfactory assistance performance. In sum, the integration of fault-tolerance control and neural supervised loop exhibits potential for HIL systems, which implements a switching mechanism for predefined state events ensuring safe command over robots, enabling long-term closed-loop stability.

HIL systems also face challenges from environmental factors. How to realize the adaptation of the robotic systems and to robustly cope with uncertainties, is another interesting Research Topic. Tendon-sheath mechanisms (i.e., cable-driven actuation or smart material actuation) have attracted widespread interests due to flexibility, safety, and dexterity. These salient features guarantee a comfortable and user-friendly human-robot-environment interaction. Shi et al. proposed an adaptive control method by describing a SMA actuator as a gray-box model. The adaptation algorithm was built upon the multi-innovation concept and incorporated a dead-zone weighted factor, aiming to reduce computational complexities and enhance robustness properties. The experimental results of a SMA actuated hand rehabilitation robot had achieved higher position tracking accuracy. Ren et al. dealt with the precise modeling of non-linear friction, and proposed a novel fuzzy control scheme for the Euler-Lagrange dynamics model of tendon-sheath mechanisms, which achieved satisfactory tracking performance and provided more accurate friction compensation. Both of these methods are model-free control and have no strict requirement for the dynamics model, which could help improve human-robot locomotion adaptations, and thus exhibit potential applications in human-robot-environment interaction within the field of HIL based neural rehabilitation training.

Results from clinical practice have demonstrated the practicality and reliability of HIL systems. Li X. et al. analyzed the effect of electromyographic feedback functional electrical stimulation (FES) on the changes in the plantar pressure of drop foot patients. This case-control study enrolled 34 stroke patients with foot drop during a 4 weeks' treatments in a Rehabilitation Center at hospital. Compared with the control group, the advantages of FES were more significantly reflected in the improvement of gait speed, the step length symmetry index, and the enhancement of propulsive force. The results indicated that integrating FES with robot is more effective than basic rehabilitation training for stroke patients with foot drop. Ying et al. dealt with the classification problem of some neurological diseases such as Parkinson's Disease and Multiple System Atrophy, and proposed a novel feature extraction framework called 3D-CAM for computer-aided diagnosis. The dataset for this study was obtained from the Neurology Outpatient Department of a hospital, covering patient data from July 2020 to August 2023. Both studies provide new ideas for diagnosis and treatment of neurological diseases by using a more general HIL concept for clinical applications.

## Author contributions

BZ: Writing – original draft, Writing – review & editing. YL: Writing – original draft, Writing – review & editing. BH: Writing – original draft, Writing – review & editing. NY: Writing – original draft, Writing – review & editing. XJ: Writing – original draft, Writing – review & editing.
